# Mohawk impedes angiofibrosis by preventing the differentiation of tendon stem/progenitor cells into myofibroblasts

**DOI:** 10.1038/s41598-022-24195-5

**Published:** 2022-11-21

**Authors:** Asma Mechakra, Junxin Lin, Yuwei Yang, Xiaotian Du, Jingwei Zhang, Paul Maswikitu Ewetse, Feifei Zhou, Enateri Alakpa

**Affiliations:** 1 Dr. Li Dak Sum & Yip Yio Chin Center for Stem Cells and Regenerative Medicine, Hangzhou, China; 2 Key Laboratory of Tissue Engineering and Regenerative Medicine of Zhejiang Province, Zhejiang University School of Medicine, Hangzhou, China; 3 Zhejiang University-University of Edinburgh Institute & School of Basic Medicine, Zhejiang University School of Medicine, Hangzhou, China

**Keywords:** Mesenchymal stem cells, Stem cells, Diseases, Medical research, Molecular medicine

## Abstract

Adult tendons heal via fibrovascular scarring with inferior biomechanical properties. Mohawk (Mkx) emerged as a pivotal actor in tenolineage commitment. However, its precise function in tendinopathy remains poorly understood. This study investigates the cellular and molecular mechanisms underlying Mkx’ role in fibrovascular healing. Human samples were collected to test fibrovascular markers. We then performed RNAseq on *Mkx−/−* mice compared to their wild type littermates to decipher Mkx regulome. We therefore sought to reproduce TSPCs transition to myofibroblasts in-vitro by over-expressing MyoD and followed by phenotypic and experimental cells’ characterization using microscopy, qRT-PCR, flow cytometry sorting, presto-blue cell viability assay and immunofluorescence. Two different in vivo models were used to assess the effect of the MyoD-expressing myofibroblasts: transplantation in the dorsal area of immunodeficient mice and in an adult Achilles tendon injury model. To prevent angiofibrosis, we tested the molecule Xav939 and proceeded with histological stainings, q-RT PCR transcriptional quantification of angifibrotic markers, mechanical tests, and immunofluorescence. Tendinopathy samples showed fibrovascular healing with decreased tenolineage phenotype. Transcriptomic analysis of *Mkx*−/− tendons revealed myofibroblast-associated biological processes. Over-expression of MyoD in WT tendon stem progenitor cells (TSPCs) gave rise to myofibroblasts reprogramming in-vitro and fibrovascular scarring in-vivo. MKX directly binds to MyoD promoter and underlies global regulative processes related to angiogenesis and Wnt signaling pathway. Blocking Wnt signaling with the small molecule Xav393 resulted in higher histological and biomechanical properties. Taken together, our data provide the first in vivo and in-vitro evidence of tendon stem progenitor cells to myofibroblasts transition and show improved tendon healing via angiofibrosis modulation, thus opening potential therapeutic avenues to treat tendinopathy patients.

## Introduction

Tendons connect muscles to bones, ensuring force transmission and therefore joint movement. Repetitive loading and overuse make tendons prone to degeneration following injuries^1^. Tendinopathies are commonly encountered in clinical practice and are associated with neo-fibrovascular tissue formation^2,3^. Adult tendons never regain their original biomechanical properties due to their poor regenerative potential and the current treatment strategies do not procure complete recovery given the lack of understanding of tendon physiopathology^4^.

The persistence of myofibroblasts is a hallmark of fibrovascular scar healing in a wide range of organs and tissues^5^. These cells have a phenotype combining both fibroblast and smooth muscle cells' characteristics^6,7^. In tendons, the cells underlying fibrovascular scarring are not fully defined.

Mkx, a key transcription factor (TF) of tenogenesis, participates decisively during development in tendon differentiation and morphogenesis by regulating type I collagen production in tendon cells^8,9^. Studies with Mkx−/− mice show that these mice exhibit reduced tendon formation throughout the body, suggesting the role of Mkx is extended during adult tendon homeostasis^10^. Moreover, in-vitro studies showed that Mkx over-expression promotes the expression of teno-related genes and safeguards tenogenic identity by preventing the expression of other cell lineages^10–13^. Mkx is substantially down regulated in human tendinopathy and the in-vivo transplantation of mesenchymal stem cell sheets expressing Mkx promoted tendon repair in a mouse model of Achilles‐tendon defect^9^. At present, no study has explored Mkx effectors during tendinopathy and the signaling cues leading to its down regulation remain to be determined. Given that tenocytes are specialized fibroblasts deriving from TSPCs/MSCs and that Mkx has a central role in maintaining tendon identity and conferring protection from aberrant cellular differentiation, we hypothesized that TSPCs may differentiate into myofibroblasts and that Mkx may have a negative control over this transition.

## Materials and methods

Detailed experimental procedures are available in the supplemental information section online.

### Human samples

Human tendon biopsies were collected from 5 different patients undergoing proximal biceps tendon tenotomy in the Second Affiliated Hospital, School of Medicine, Zhejiang University. Surrounding, non-lesioned tissues were used as controls.

### Mouse TSPCs isolation and cell culture

Mouse TSPCs were isolated from 2 weeks old *Mkx*−/− and wild type (WT) mice tail tendons. Husbandry, handling, and sacrifice of the mice prior to TSPCs isolation were carried out according to the guidelines of Zhejiang university animal center regulations. Mice were euthanized by intraperitoneal injection of a lethal dose of sodium pentobarbital (120 mg/kg). Cells at passages 1–5 are used for experiments.

### High throughput RNA sequencing

Total RNA of tail tendons was extracted (n = 3) using TRIzol reagent (Takara, Japan) and cDNA library was prepared using the Ion Total RNA-Seq Kit v2. RNA sequencing was performed on Ion Proton system. Clean reads were obtained from the raw reads by removing the adaptor sequences, reads with > 5% ambiguous bases (noted as N) and low-quality reads containing more than 20 percent of bases with qualities of < 13. The clean reads were then aligned to mouse genome (mm9) using the MapSplice program (v2.1.6). We applied EBseq algorithm^14^ to filter the differentially expressed genes under the following criteria: (1) Fold Change > 1.5 or < 0.667; (2) FDR < 0.05.

### Myofibroblast differentiation

Neonatal TSPCs were transfected at 60–70% confluence with *MyoD* expressing vectors (CMV-*MyoD*, a gift from Andrew Lassar, Addgene plasmid # 8398) or with empty vectors (controls, CMV-*MyoD* restricted using EcoRI and ligated) using lipofectamine 2000 reagent (Invitrogen, USA) according to the manufacturer’s recommendations. Cells were cultured in L/G DMEM medium that was changed every 2 days.

### Myofibrobalsts transplantation in nude mice

To test *MyoD*-expressing myofibroblasts effect in-vivo, we transplanted 10^6^ cells mixed with Gelatin methacryloyl; GelMA^15^, a biomaterial capable of fast photopolymerization under UV irradiation using an improved method^16^, into immunodeficient mice (9 weeks old males, n = 5). Longitudinal skin incisions of about 2 cm in length were made on the dorsal surface. Cells/biomaterial mix were loaded dropwise into the subcutaneous pockets we made in the muscle adjacent to the incision (UV rays were used for polymerization, one transplant /mice). Incisions were closed with surgical sutures. The same procedure has been applied to the control group (n = 5, transplanted with TSPCs transfected with the empty vector). Samples were collected 2 weeks after transplantation.

### Dil staining and transplantation in adult injured mice

To further explore whether myofibroblasts contribute to fibrosis and neo-angiogenesis in vivo, we transfected TSPCs isolated from WT mice with *MyoD* vector (or empty vector for the control group) and stained both the myofibroblast and the control groups with Dil staining (1,10-dioctadecyl-3,3,30,30-tetramethylindocarbocyanine perchlorate, Beyotime Institute of Biotechnology, China). Each group of mice (all male, n = 6/group) was subjected to Achilles tendon hemisection and cells were transplanted as described above. Tendons were collected 28 days after injury.

### Angiogenesis modulation in-vivo

To modulate angiogenesis in-vivo during mice adult fibrovascular scar healing, injured Mkx^+/-^Achilles tendons from 8 months old mice were treated with Xav939 (4 µM, Selleck Chemicals, USA) to inhibit Wnt/β-Catenin pathway. A slow-release biomaterial technology was used as a biological glue. Xav939/GelMa mix was loaded dropwise into the injury site concomitantly to UV irradiation prior to suturing. Control group received GelMa alone. Samples were collected at two time points: at day 28 or day 52 after surgery.

### Biomechanical properties testing

Achilles’ tendons isolated from *Mkx*^+/−^ mice (non-treated; control group or treated; Xav939 group) were clamped from the calcaneus bone and myotendinous junction. Tissues were preloaded to 1 N for 5 loops followed by a speed of stretch of 10 mm/min using the single column electromechanical testing system Instron 5944. The maximum force, stiffness and yield elongation were measured. N = 7 independent samples were tested per group.

### Statistical analysis

Data are presented as mean values ± SEM. Statistical analyses of quantitative experimental data are performed using the unpaired Student t-test. p-value < 0.05 was considered statistically significant. All the experiments have been independently reproduced at least 3 times.

GraphPad Prism 6 software (GraphPad Software) was used to analyze quantitative data.

### Supplementary materials

This file contains supplementary information on methods, sypplementary figures (S1 and S2), a supplementary table listing the primers used for qRT-PCR analysis and supplementary references.

### Ethics approval and consent to participate

Animal experimentation procedures comply to the rules of Zhejiang University Institutional Animal Care and Use Committee and have been approved by the Ethics Committee of the School of Medical sciences of Zhejiang University (reference number: 12053). This study complies with ARRIVE guidelines. Human tendon biopsies collection was approved by ethics committee of the Second Affiliated Hospital, School of Medicine, Zhejiang University (approval number: 2017-280 for use of surgical waste). The study conforms to the principles of the Declaration of Helsinki and participants gave informed consent.

## Results

### Fibrovascular markers are up regulated in human tendinopathy

**Figure 1:**
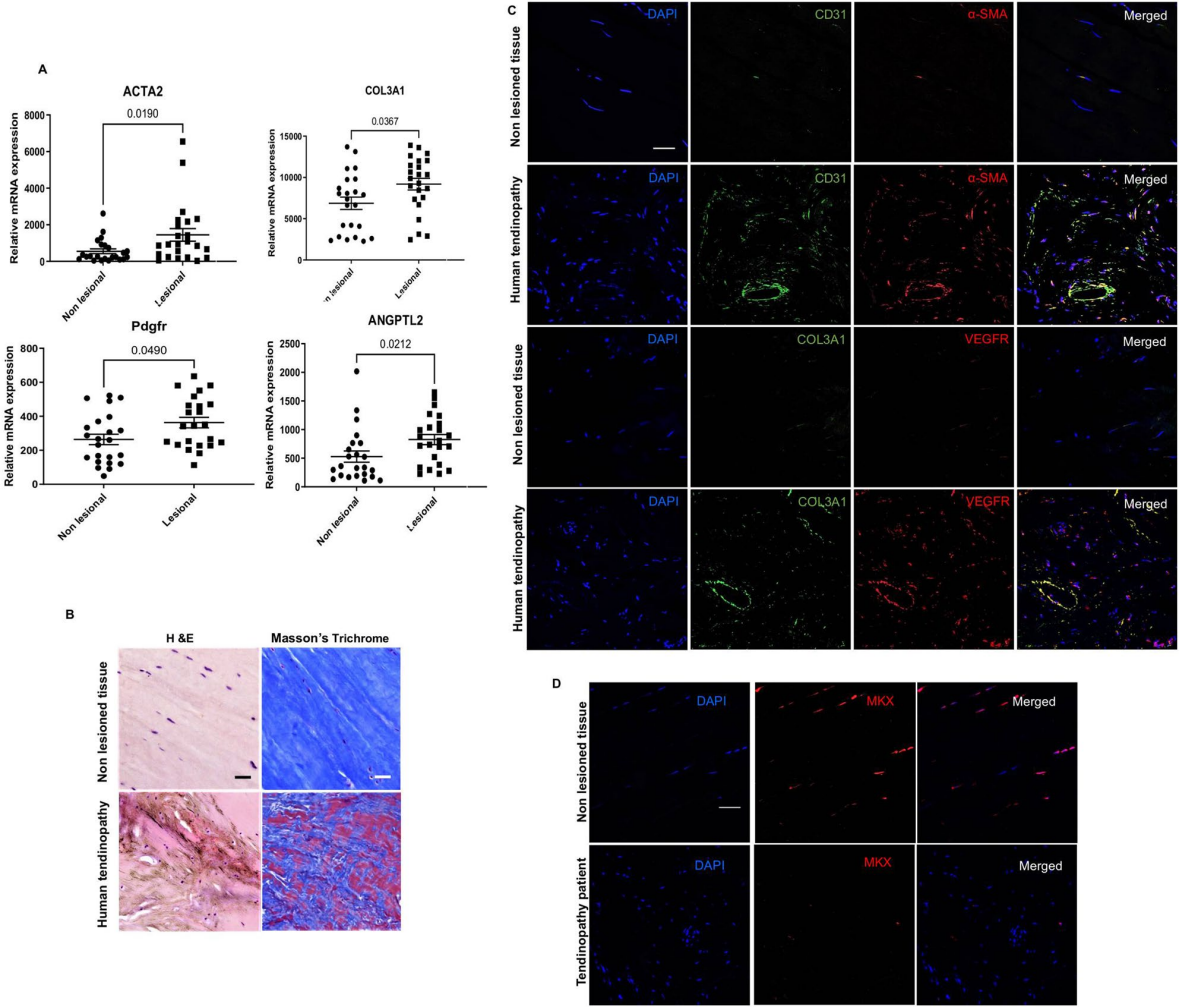
Fibrovascular scarring in human tendinopathy. (**A**) Acta2, Col3a1, CD31, Angptl2 and Pdgfr gene expression profiling from GEO datasets of human tendinopathy and surrounding non-lesioned tissues (n523, Affymetrix platform, profile reference: GSM1310181). (**B**) Histological staining of diseased proximal biceps tendon sections or non-lesioned control tissues (*n* = 5). Scale bar, 50 μm. (**C**) Representative immunofluorescence images (n = 5) of human proximal biceps tendon sections stained for α-SMA/VEGFR2, CD31/Col3a1 or DAPi. Scale bars, 40 μm. D. Representative MKX expression in tendinopathy specimens from 2 two different patients. DAPI (blue), MKX (red). Scale bar, 40 µm.

Most of the studies describing fibrovasculogenesis are anatomo-pathological. To our best knowledge, only 2 studies have used quantitative and semi-quantitative methods to assess the expression of a well-known angiogenic marker, VEGF, in human diseased Achilles tendons^17,18^. We therefore sought to investigate key fibrovascular markers in human tendinopathy. On the one hand, we screened publicly available data on human chronic tendinopathy^19^ from gene expression omnibus (GEO) datasets. The fibrotic markers *ACTA2, COL3A1 and PDGFRB*, in addition to the angiogenic marker Angptl2 were found to be up-regulated (Fig. 1A). On the other hand, human proximal biceps tendinopathy specimens showed a highly degenerative aspect (Fig. 1B), increased signal forf α-SMA, COL3A1, CD31 and VEGF (Fig. 1C) and decreased MKX signal (Fig. 1D).

### Mohawk prevents fibrovascular scar formation

**Figure 2:**
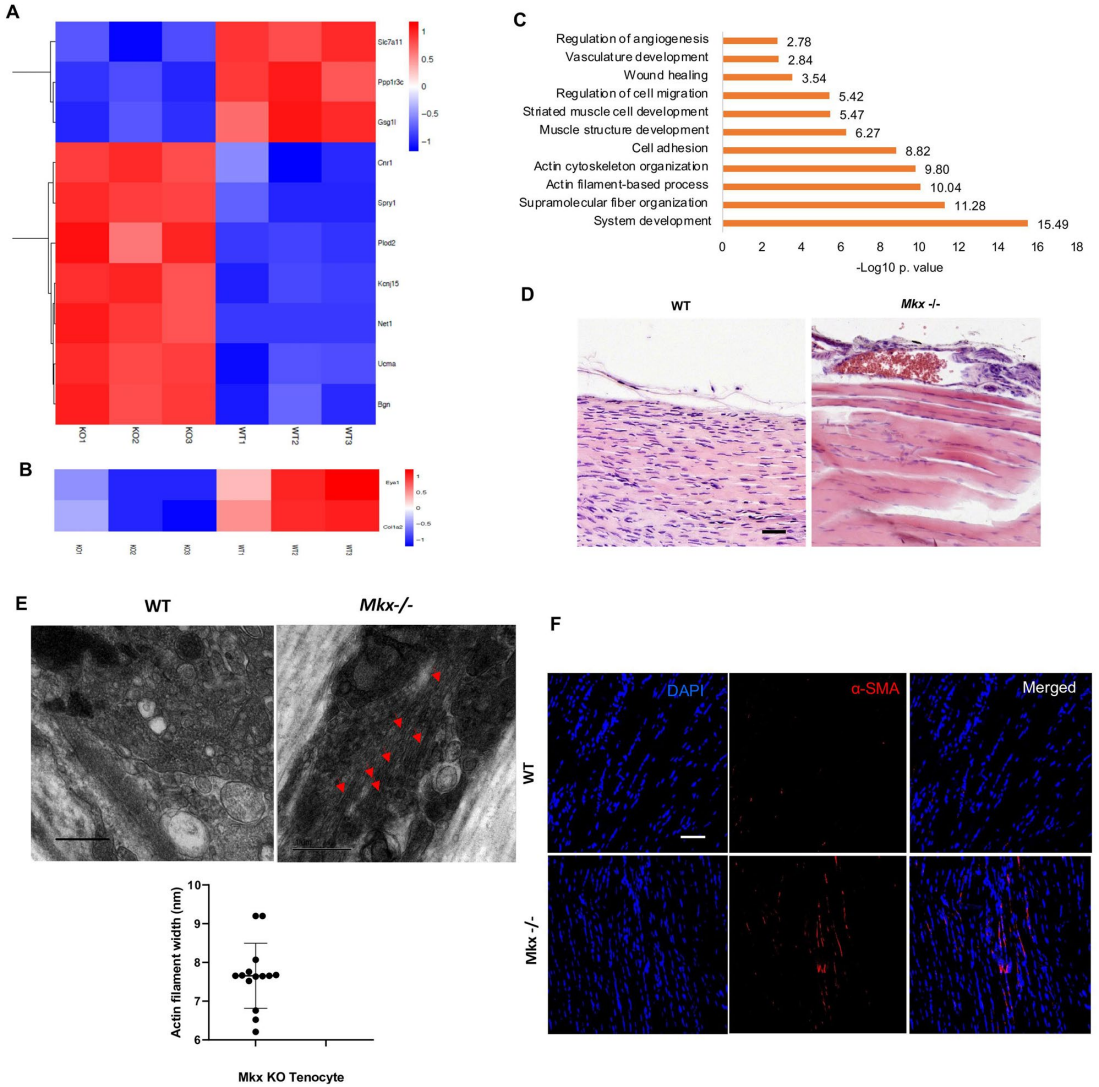
Molecular signature of the Mkx−/− mice. (**A**) Heat map of the top 10 DURGs. **(B**) Differentially down-regulated teno-related genes. (**C**) DURGs GO biological processes. (**D**) Histological confirmation of the *Mkx*−/− angiogenic phenotype (H&E staining, n = 3). Scale bars, 50 μm. (**E**) Transmission electronic microscopy (TEM) micrographs representing the cytoplasm of Achiles tendons resident cells (WT and *Mkx−/−,* n = 3). Red arrows represent actin-like filaments. Scale bars, 1 μm. (**F**) α-SMA expression in the Mkx−/− vs. WT AT (2 weeks old, N = 3 per group).

High-throughput RNA-Seq was performed to explore the *Mkx* transcriptome. *Mkx*−/− mice showed 923 differentially expressed genes in comparison to their WT littermates. Net1 and Plod2, 2 fibrovascular related genes were among the top 10 differentially up-regulated genes (DURGs, Fig. 2A). Eya1 and Col1a2, two teno-related genes were down regulated in the *Mkx*^−/−^ mice (Fig. 2B). “Actin-filament”, “cell adhesion”, “wound healing” and “Angiogenesis” were among the most enriched biological processes **(**angiogenesis was also confirmed at the histological level**, **Fig. 2C and D). Moreover, pathway analysis of *Mkx*^−/−^ differentially up-regulated transcripts using KEGG database showed “focal adhesion” (16 implicated transcripts), and “Wnt signaling pathway” (7 implicated transcripts, including Fzd1 and Wnt2) were among the most enriched pathways. Some of the *Mkx*^*−/−*^ resident cells (Fig. 2E) exhibited a higher signal for α-SMA (Fig. 2F). To bring stronger evidence regarding *Mkx* negative regulation of angiofibrosis in-vivo, we applied a neonatal regeneration model to *Mkx*^−/−^ neonates*.* WT neonatal tendons had the appearance of non-injured tendons and were α-SMA negative. In the *Mkx*−/− mice, injured tissues exhibited robust fibrosis in addition to marked vascularity as shown by H&E and Masson trichrome staining (Fig. S1A). Immunofluorescence of paraffin-embedded sections showed COL3A1 and α-SMA protein up-regulation (Fig. S1B). These data show the importance of *Mkx* in safeguarding tendon identity by preventing angiofibrosis.

### MYOD reprograms TSPCs to myofibroblasts in-vitro and promotes angiofibrosis in-vivo

**Figure 3:**
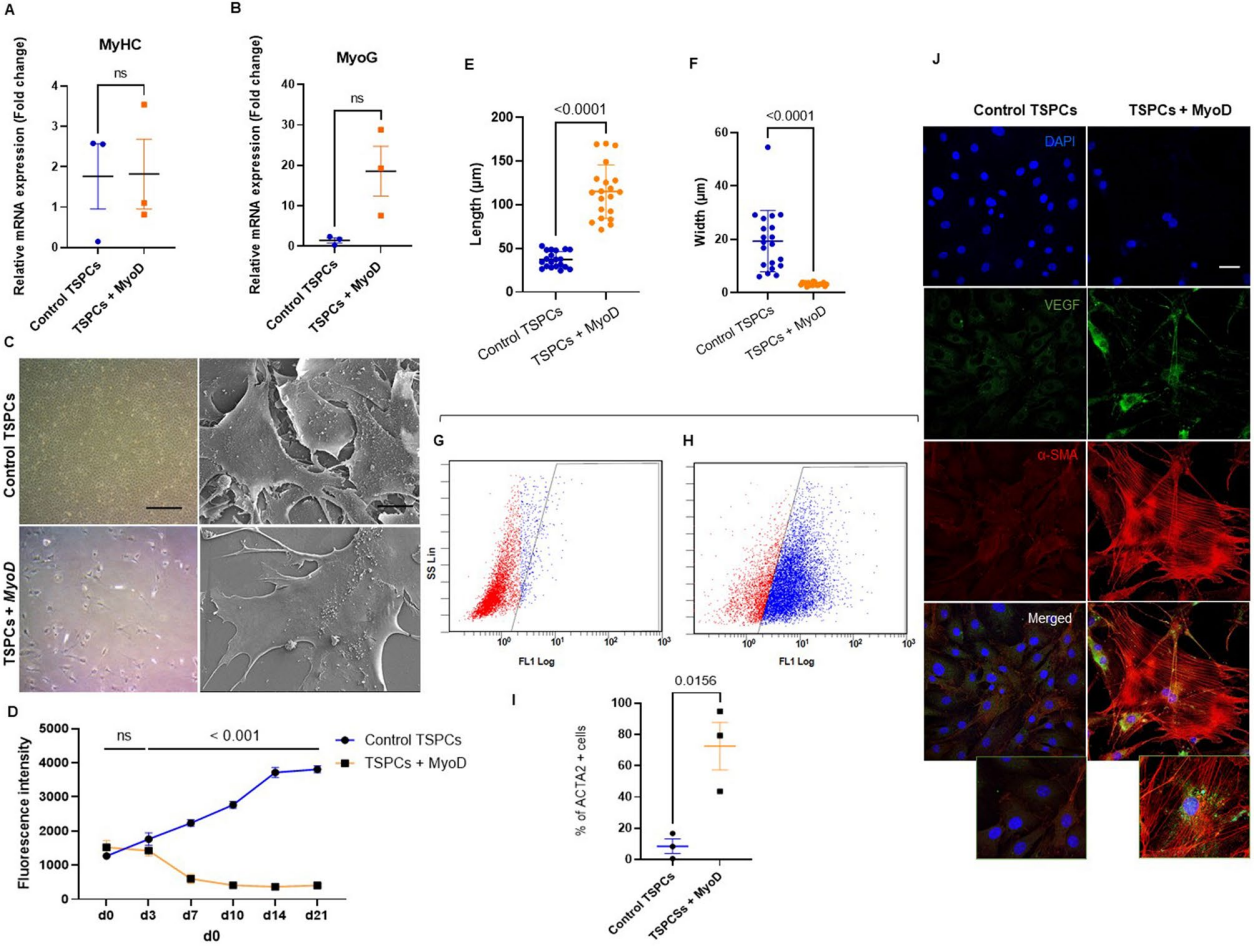
MyoD induces TSPCs to myofibroblasts reprogramming in vitro. (**A**) and **(B**) Acta2 and Col3a1 qRT-PCR quantification comparing 3 conditions: “control TSPCs”, “MyoD-expressing TSPCs (14 days after transfection)” and “TSPCs + MyoD + Xav939 treatment”. N = 3. (**C**) Representative morphological change 14 days after MyoD expression under inverted (scale bar, 50 μm) or scanning electron microscope (scale bar, 25 μm). (**D**) PrestoBlue cell viability assay of *MyoD*-expressing or control TSPCs. ***p < 0.001. (**E**) Cellular length comparison and (**F**) width (Control vs. MyoD-expressing TSPCs 14 days after transfection) using image J software, 20 microscopic fields, **p < 0.01. (**G**) FACS gating strategy for control TSPCs and (**H**) myofibroblasts. (**I**) FACS based quantification of ACTA2 + cells. *p < 0.05. N = 3. (**J**) Representative immunofluorescence images of MyoD-expressing TSPCs *vs*. control TSPCs stained for VEGF (green) and α-SMA (red). N = 3. Scale bar, 20 μm.

Our transcriptomic data and subsequent histological characterization suggest the presence of myofibroblasts. Given that tenocytes derive from mesenchymal stem cells, which are multipotent stromal cells that can differentiate into multiple cell types, we hypothesized that TSPCs may differentiate into myofibroblasts. We used the transcription factor *MyoD* to induce the transition given its previous implication in myofibroblast fate transition, fibrosis and angiogenesis. The workflow is illustrated in Fig. S2A. One week after MyoD transfection, cells showed filopodia formation (Fig. S2B). At d14, cells did not exhibit neither the multinucleated phenotype characteristic of skeletal muscle cells nor mRNA up-regulation of a selection of markers (Fig. 3A and B, Dmd was not expressed in both groups). However, limited cell growth (d7, F = 2234.94 ± 94.88 vs. 601.91 ± 135.15, p < 0.001, for control TSPCs and MyoD-expressing TSPCs respectively, as measured by presto blue cell viability assay) accompanied with significant increased length and decreased width was observed (Fig. 3C–F). The myofibroblast marker α-SMA was dramatically up-regulated (% of ACTA2 + control TSPCs: = 8.56 ± 379 vs. 72.57 ± 12.38 for TSPCs + MyoD, p < 0.01) (Fig. 3G–I). Additionally, the angiogenic marker VEGF was highly expressed in the α-SMA + myofibroblasts (Fig. 3J). These data suggest a remarkable plasticity of TSPCs to transition into myofibroblasts.

To test the effect of MyoD-expressing myofibroblasts in vivo, these cells, 14 days after transfection, were transplanted subcutaneously into the dorsal surface of immunodeficient mice using Gelatin methacryloyl (GelMA) to compare with control TSPCs. Two weeks after transplantation, highly organized tendon-like structures were visualized in the control TSPCs’ transplanted group (Fig. S2C, upper panel). The difference in collagen deposition was not significant (Fig. S2D) and minimal CD31 and α-SMA fluorescence was detected at the transplantation area (Fig. S2E, upper panel). In contrast, MyoD-expressing myofibroblasts led to fibrovascular tissue formation (Fig. S2C, lower panel) and positive signal corresponding to CD31+/α-SMA+ was visible at the transplantation area (Fig. S2E, lower panel).

Second, we transfected WT TSPCs with *MyoD* and stained them with Dil 2 weeks after transfection to track their localization in-vivo. We then injured adult mice Achilles tendons and transplanted Dil stained cells at the injury site using the same carrier (GelMA). *Myod*-expressing myofibroblasts not only promoted fibrovascular scar formation but also participated to neo-vascularization as showed by Dil staining in the cells bordering structures resembling the vessels (Fig. S2F).

### MKX regulates MyoD, angiogenesis and Wnt signaling pathway

**Figure 4:**
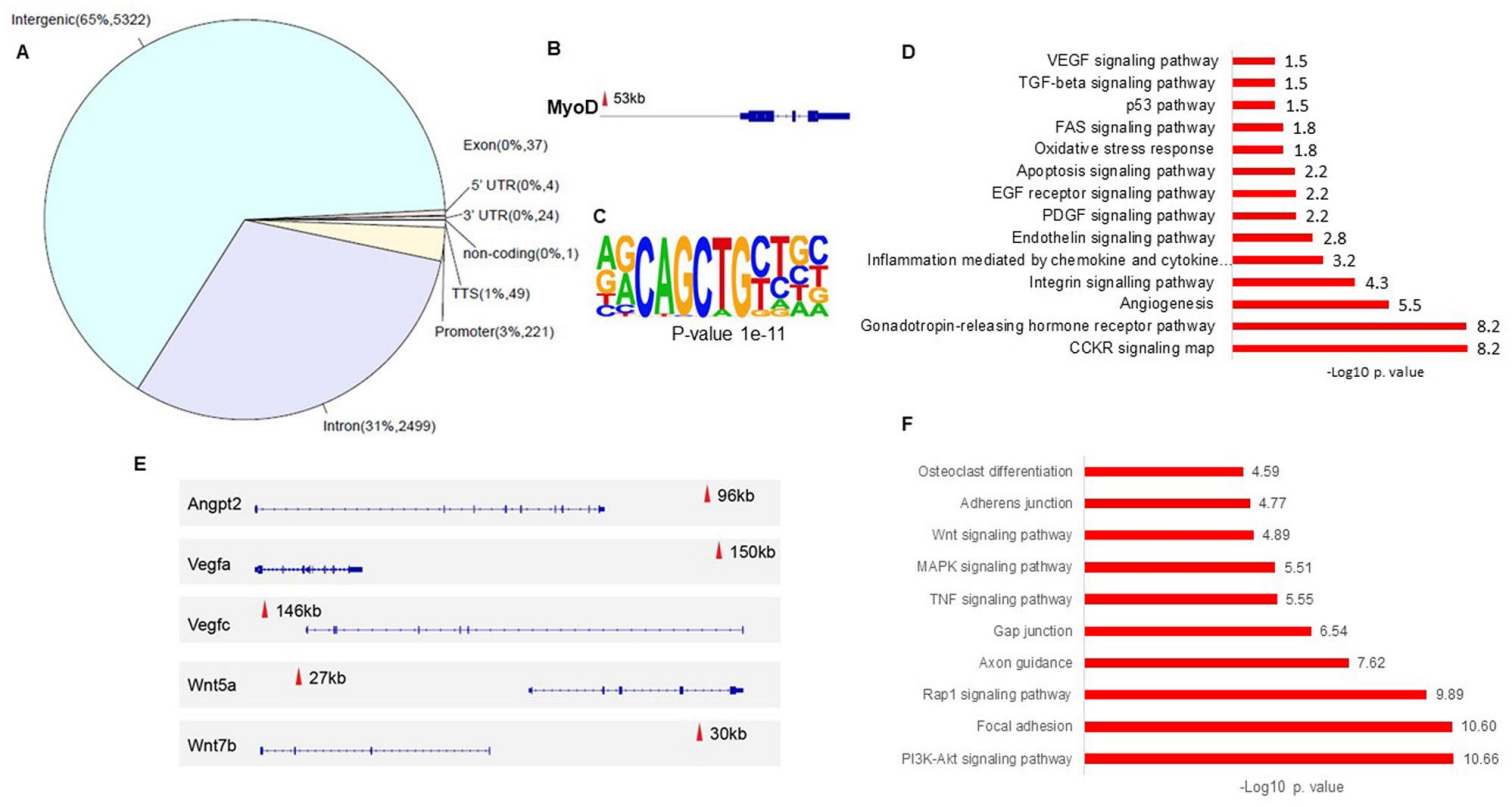
MKX CHIPseq analysis. (**A**) Genomic distribution of MKX binding sites. (**B**) Reanalysis of publicly available ChIP-seq data of tendon-derived cells revealed that *MyoD* is a putative target of Mkx. (**C**) De novo motif analysis revealed a MyoD binding motif. (**D**) GO biological processes analysis. (**E**) Set of tracks representing Vegfa/c, Angpt2, Wnt5a, and Wnt7b peaks. (**F**) KEGG pathway analysis.

In a study by Chuang HN et al., MKX represses and directly binds to *MyoD* promoter using zebrafish and mouse constructs in a myoblast cell line^20^. We further analyzed MKX CHIPseq publicly available data of rat tenocytes^13^ (Fig. 4A) and found a peak near *Myod* (intergenic region situated between 102343729–102343877 of chromosome 1) (Fig. 4B). Using *Homer* software for de novo motif discovery, we detected one enriched motif corresponding to *Myod* (Fig. 4C). “Angiogenesis” was one of the top 3 enriched GO terms with 70 related genes including *Vegfa*/c, *Angpt2*, *Wnt5a* and *Wnt7b,* in addition to other vascular-related terms (“Endothelin signaling pathway” and “VEGF signaling pathway” (Fig. 4D and E) and Wnt signaling among the most enriched pathways (Fig. 4F).

### Blocking angiogenesis suppresses adult fibrovascular scarring

**Figure 5:**
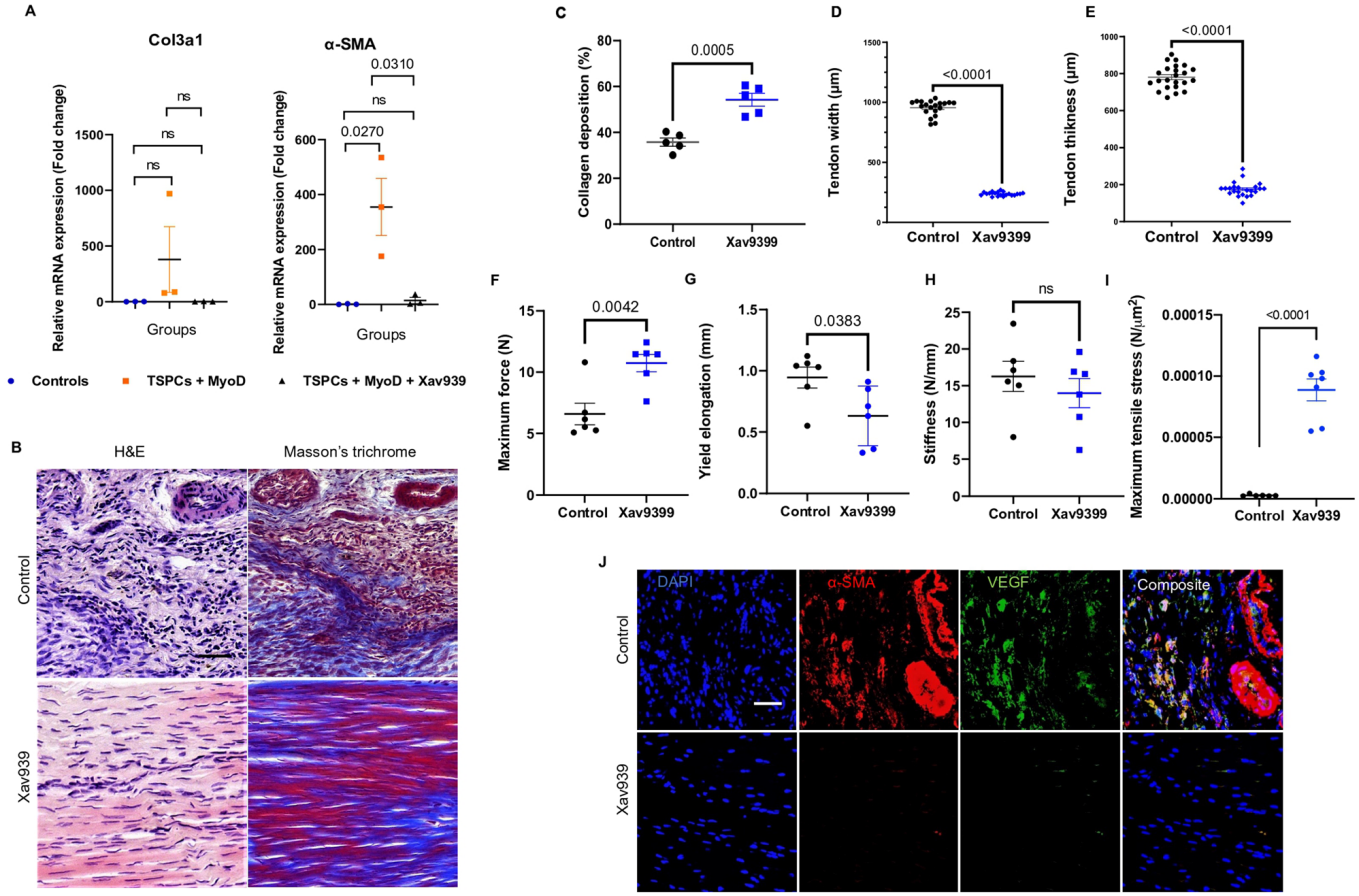
In-vivo Wnt signaling modulation during adult tendon healing. (**A**) qRT-PCR Acta2 and Col3a1 quantification comparing 3 conditions: (1) control TSPCs (2) MyoD-expressing TSPCs (3) TSPCs + MyoD + Xav939 treatment. N = 3. (**B**) Representative H&E and Masson’s trichrome staining images of control and Xav939-treated tendons d28 after injury. N = 6 tendons/group. Scale bar, 40 μm, and (**C**) the corresponding Collagen deposition quantification**. **(**D**) Width quantification of control and Xav939-treated Achilles tendons (20 zones along 5 tendons/group). (**E**) Tendon thickness of control and Xav939-treated tendons (20 zones from 5 tendons/group). (**F–I**) Maximum force, yield elongation, stiffness and maximum tensile stress properties in the control and Xav939-treated groups d52 after injury. N = 6. Representative immunofluorescence staining images of α-SMA and VEGF.N = 5 tendons/group. Scale bar, 40 μm.

Based on our findings, we hypothesized that blocking neoangiogfibrosis could prevent adult fibrovascular scarring. We therefore used Xav939, a potent Wnt\β-catenin inhibitor^21^ to test our hypothesis. We first assessed mRNA expression of Col3a1 and α-SMA after MyoD transfection in mouse TSPCs and found that the small molecule restored the expression of α-SMA mRNAs to levels close to the control (Fig. 5A).

Secondly, the effect of Xav939 was ascertained using an adult injury model. The results were gratifying as they demonstrated a phenomenal effect on adult tendon regeneration 28 and 56 days after injury. Consistent with histological analysis showing well organized collagen fibers in the Xav939-treated group in contrast to fibrotic and disorganized collagen fibers in the non-treated group (Fig. 5B and C). The tendon general aspect of the Xav939 treated group was also significantly improved (Fig. 5D and E). Mechanical properties were quantified 52 days after injury. Xav939 group showed higher maximum force and lower yield elongation but no significant difference in stiffness was observed (Fig. 5F–H). Maximum tensile stress was also higher in Xav939 group (Fig. 5I). Fibrosis and angiogenesis markers α-SMA and VEGF were dramatically decreased in Xa939 group in comparison to the control group (Fig. 5J).

## Discussion

Despite considerable advances in researching the cell and molecular mechanisms governing tendon physiology and healing, the mechanisms underlying fibrovascular scarring need to be investigated further. Using clinical samples, we confirmed this histopathological feature in proximal biceps tendinopathy. By combining mouse models, in-vivo and in-vitro experiments, we showed that the transcription factor MKX maintains tendon signature and is a negative regulator of tendon neo-angiofibrosis.

RNAseq data analysis indicated actin cytoskeleton remodeling and focal adhesion, which have both a role in scar formation and myofibroblast-mediated contraction steps^22–24^, are among the most enriched up-regulated biological processes and pathways.

When *MyoD* was over-expressed in WT neonatal TSPCs, *MyoD* induced myofibroblast transition. We demonstrate that these cells were sufficient to trigger both vascular remodeling and fibrosis when transplanted either on nude mice or on WT mice with injured Achilles tendons. Mayer, D. C. and Leinwand, L. A. have previously reported the activation of muscle gene programs, including MyoD and muscle structural proteins, in two myofibroblast cell lines from kidney mesangial cells and liver stellate cells^25^. In a human lung fetal model, *MyoD* was required for myofibroblastic differentiation in a reversible fashion^26^. MyoD^+^ cell subpopulation has been shown to regulate wound response and give rise to myofibroblasts in the lens epithelium^27^ and MyoD^+^ and NOGGIN^+^ cells containing α-SMA contribute to posterior capsule opacification of the lens following cataract surgery^28^. In addition, the combination of 3 transcription factors (Myocd, Mef2C and Gata6), with the transactivation domain of MyoD efficiently reprogrammed mouse embryonic fibroblasts to smooth muscle-like cells^29^. *MyoD* mRNA has been found to be up-regulated in the trunk somites of *Mkx* Zebrafish morphants and the use of an immortalized mouse myoblast cell line (C2C12) showed that Mkx negatively regulates myoD expression^20^. MyoD temporal expression in relation to *Mkx* during tendon healing remains unexplored and deserves future investigations.

As muscle related terms were enriched in the RNAseq data of the KO mice, we checked MyoD expression in the Mkx−/− mice, but we could not reach unequivocal conclusions as the qRT-PCR and the western blot results were not consistent (Figure below). This is likely linked to the highly dynamic nature of transcription factors in general^30^, and MyoD is no exception^31,32^. We are aware that the temporality of MyoD expression in relation to Mkx deserves to be studied, but we deem this beyond the scope of our work and we chose to use MyoD as a tool^26^ for myofibroblast transition.

In addition to an increased vascularity around the fibrotic area in the injury/regeneration models, our results also showed angiogenesis as one of the most enriched biological processes according to RNA sequencing of the KO mice and MKX CHIP sequencing data analysis. Neovascularization has long been reported as an underlying condition in lung fibrosis. In kidney or adipose tissue, it is implicated in different pathological contexts, and is thought to participate to fibroproliferation^33–35^. This is supported by several studies demonstrating angiogenesis blocking as an efficient strategy to prevent fibrosis^36^.

The “continuum model” proposed by Cook and Purdam in 2009 is one of the current dominant theories of tendinopathy. According to this model, tendinopathy ranges in 3 categories: from reactive tendinopathy, tendon disrepair to degenerative tendinopathy^37^. Surgery remains the last treatment option, after exhausting all other conservative means of care^38^ and concerns late tendon disrepair and degenerative tendinopathy. Both late disrepair and degenerative stages present fibrovascular characteristics^37^.

If the participation of resident cells (from epithelial or fibroblastic origins) to the myofibroblast population in fibrotic diseases has been established^5,39,40^, the participation of endothelial cells to the myofibroblast pool has on the other hand been a matter of debate. Surprisingly, when we transplanted *MyoD*-expressing myofibroblasts in-vivo, Dil staining was observable at the level of few cells constituting blood vessels, suggesting an opposite phenomenon: a myofibroblast to endothelial transition. Lemoinne et al.^41^ reported a similar phenomenon in the liver where portal myofibroblasts participate to hepatic vascular remodeling by releasing VEGFA microparticles and integrating the newly formed vessels as mural cells. Our *prima facie* finding in tendon necessitates further robust in-vivo evidence and a complete study per se.

Injured Achilles tendon treatment for angiogenesis modulation with a selective Wnt/β-Catenin inhibitor, the small molecule Xav939, resulted in a phenomenally improved regeneration. The canonical Wnt/β-catenin pathway is involved in a range of processes related to angiogenesis, vascular remodeling and fibrosis^42–44^. In the study of human tendinopathy by Jelinsky et al.^19^, several genes involved in Wnt signaling were among the most enriched transcripts. β-catenin, a master molecule of the canonical Wnt/β-catenin signaling, is also up-regulated during human tendinopathy (on reanalysis of Jelinsky et al., publicly available datasets shows an increased expression of β-catenin, data not shown). In another study, in addition to β-catenin up-regulation, Wnt/β-catenin signaling activation was correlated to Scx, Mkx, and Tnmd suppression during adult rat Achilles tendon injury^45^.

## Conclusions

Our findings demonstrate *Mkx* essentiality to preserving tendon integrity and impeding from fibrosis and neo-vascularization during wound healing. MyoD over-expression provokes TSPC to myofibroblast transition. And lastly, Xav939, a potent Wnt/β-Catenin pathway inhibitor to modulate angiogenesis leads to a markedly improved healing, providing a rationale for the use of Wnt/β-Catenin modulators for the treatment of tendinopathy.

## Supplementary Information

Supplementary Information.

## Data Availability

RNAseq data have been deposited in GEO database under the reference GSE102926 (https://www.ncbi.nlm.nih.gov/geo/query/acc.cgi?acc=GSE102926).
