# Supplementary material for: Mohawk impedes angiofibrosis by preventing the differentiation of tendon stem/progenitor cells into myofibroblasts
Source: Sci Rep. 2022 Nov 21;12:20003. doi: 10.1038/s41598-022-24195-5 (PMC9678895; doi:10.1038/s41598-022-24195-5)
Supplement: Supplementary file 1 — Supplementary Information. [file 41598_2022_24195_MOESM1_ESM.docx]

**SUPPLEMENTAL information**

# Mohawk Impedes Angiofibrosis by Preventing the Differentiation of Tendon Stem/Progenitor Cells into Myofibrobalsts

# Asma Mechakra, PhD ^1*^, Junxin Lin, MSc, Yuwei Yang, B.S, Xiaotian Du, MSc, Jingwei Zhang, PhD , Paul Maswikitu Ewetse, MD, Feifei Zhou, MSc, Enateri Alakpa.

***Corresponding author**:

Dr. Asma Mechakra

Center for Stem Cell and Tissue Engineering, School of Medicine, Zhejiang University, 866 Yu Hang Tang Road, Hangzhou 310058, China.

E-mail: asma.mechakra@gmail.com

**Index**

**Supplementary methods 3**

**Supplementary Figures** 7

**Supplementary Figure 1.** Fibrovascular scar formation study during neonatal regeneration 7

**Supplementary Figure 2**. Myofibroblasts promotion of fibrovascular scarring *in vivo* 8

**Supplementary tables**  9

**Table S1.** Primers used for qRT-PCR analysis 9

**Supplementary references** 10

**Supplementary methods**

**Mouse TSPCs isolation and cell culture**

Tendon tissues were enzymatically treated 3-4 hours with collagenase type I in Low Glucose Dulbecco's modified Eagle's medium (low glucose DMEM, Gibco, USA) supplemented with 10% fetal bovine serum (FBS, Invitrogen, USA) and 1% penicillin / streptomycin (Gibco). Cells were then pipetted every 30 min. When the cells became individualized, complete low glucose DMEM was added to block the enzymatic digestion and cultured overnight at 37°C and 5% CO_2_. The following day, cell suspension is centrifuged at 1500 g for 3 min and suspended in fresh culture media.

**Neonatal injury model**

*Mkx*^-/-^ and wild type neonatal mice were anesthetized by intraperitoneal injection of pentobarbital (1% weight/volume, 80mg/kg, n=3 per group). A complete transection was made in the right hind limb Achilles tendon. Wild type mice served as controls. Animals were sacrificed d28 after injury and tendons were collected.

**Myofibroblast Differentiation**

First, we used an induction medium containing high glucose DMEM supplemented with 5% horse serum and 1% penicillin/streptomycin to investigate myogenic differentiation. As we found that TSPCs do not go through this lineage, we subsequently exclusively used L/G DMEM for all our cultures and experiments.

**Quantitative Real Time Polymerase Chain Reaction (qRT-PCR)**

Total RNA was extracted from all our samples (control TSPCs or *MyoD*-expressing TSPCs) using TRIzol according to the manufacturer’s recommendations. We reverse transcribed 0.5 µg of total RNA (Cinbiotech, China) and performed quantitative real-time PCR (q-RT PCR) analysis using SYBR Green Supermix (Takara, Japan) using the CFX96 Touch™ Real-Time PCR Detection System (Biorad, USA). Primers **(Table 1)** were designed using either the NCBI primer designing tool (<https://www.ncbi.nlm.nih.gov/tools/primer-blast/>) or PrimerBank (https://pga.mgh.harvard.edu/primerbank/). The copy numbers of each gene were determined by ΔΔCT method (Livak and Schmittgen, 2001). The copy numbers of *β-Actin* were used as internal controls. Data were normalized as expression relative to the respective wild time measure ± standard error of mean (SEM). N=3.

**PrestoBlue Cell Viability Assay**

The PrestoBlue assay, a ready-to-use preparation, has been used according to the manufacturer’s protocol (ThermoFisher Scientific, USA). The cells were seeded at 4 × 10^4^ cells/well in a 24-well plate. Just prior MyoD transfection, cell viability was assessed for time “d0”. Four other time points were subsequently investigated (d3, d7, d14 and d21). At each time point, cells were washed with PBS and incubated with 10% of PrestoBlue reagent in complete medium. Cell viability was then evaluated by fluorescence spectroscopy. The fluorescence was measured 30 min after incubation using the multi-mode microplate reader SynergyMx M5 with excitation and emission wavelengths of 570 and 600 nm, respectively.

**FACS**

Fluorescence-activated cell sorting (FACS) was used to detect ACTA2+ cells (Cytomic FC 500MCL). Both controls (WT TSPCs) and *MyoD*-transfected TSPCs were simultaneously treated 30 min with the rabbit polyclonal primary antibody targeting α smooth muscle actin (5µg/ml, Abcam, ab5694, United Kingdom) and the secondary antibody Alexa Fluor 488 (Invitrogen, 1:1000) then washed 3 times with PBS. We used Beckman Coulter CytoFLEX LX flow cytometer and CytExpert for data analysis.

**Transmission electronic microscopy**

Achilles tendons were isolated from WT and Mkx-/- mice (n=3) and prepared according the standard TEM protocol. Briefly, the samples were fixed with 2.5 % buffer glutaraldehyde then subjected to 3 times10 min PBS (potassium phosphate buffer) washes. They were then post-fixed in 2% osmium tetroxide in 0.1 M cacodylate buffer for 1.5 h at room temperature and passed through a graded series of ethanol and acetone solutions and infiltrated with resin. Longitudinal ultra-thin sections were realized using an ultramicrotome and collected on copper grids. The samples were examined using a Spirit 120kV (Fei Tecnai, USA) TEM operating at 60 kV.

Collagen fibril diameters of the Xav 939-treated adult mice vs. GelMa group have been assessed using the same protocol but cross-sections have been realized rather than longitudinal sections.

**Scanning electron microscopy**

TSPCs transfected with *MyoD* or control vectors grown on coverslips were gently washed with 0.5M PBS at pH 7.2 and fixed in 2.5% glutaraldehyde solution at 4 °C for at least 12 hours. Samples were then washed with 0.1 M phosphate buffered saline (PBS) and subjected to dehydration through series of ascending grades of ethanol for 15 min each. After 3 hours air drying, samples were vacuum coated for 5 min and placed on aluminum stubs for observation on Nova NonaSEM 450 (FEI, USA).

**Histology and Immunostaining**

Achilles tendons harvested from WT or Mkx-/- mice (injured or non-injured) were immediately fixed in 10% (volume/volume) neutral buffered formalin. The samples were dehydrated in successive baths of alcohol gradient, cleared, and embedded in paraffin blocks. Histological sections (7µm) were made using a microtome and primary antibodies anti α-SMA anti-COL3A1; anti-VEGFR2, Cell signaling, D5B1, USA; anti-CD31, ab222783, Abcam), then incubated overnight after blocking 20 min at room temperature with 1%BSA in PBS and heat-activation (by immersion in boiling 10 mM Sodium Citrate bufferpH 6.0, 2 times 10 min each). Appropriate secondary antibodies were used prior to confocal microscopy.

Differentiated cells (TSPCS to myofibroblasts) were cultured 2 weeks on rat tail tendon collagen-coated coverslips (Sigma, C3867-1VL, USA) and fixed in 4% paraformaldehyde then blocked with 0.1% Bovine Serum Albumin in PBS for 1hour at room temperature. Prior to primary (4°C overnight) and secondary (2 hours at room temperature) antibodies’ incubation, the samples were permeabilized with Saponin 0.01% (Sigma, USA). Nuclei counter stain were made by adding 4′, 6-diamidino-2-phenylindole (DAPI) directly to the mounting media. The cover slips were observed with a confocal microscope (Olympus BX61, Japan).

Samples used for the neonatal tendinopathy model at d28 after injury were subjected to similar procedure.

Human tendon biopsies were collected following standardized tissue banking procedures. After rinsing with PBS, samples were placed in sterilized bags, transported in dry ice and frozen at -80 °C. Biopsies were fixed in 4% paraformaldehyde for 24 hours, paraffin embedded then processed to obtain 5 μm sections. In addition to immunofluorescence, hematoxylin and eosin (H&E) and Masson’s trichrome staining have been performed according to standard procedures to examine the general histological appearance of the tissues.

**Database Knowledge**

Gene ontology (GO) analysis was performed to determine the most enriched biological processes of the differentially expressed gene. GO annotations were downloaded from GO ontology database (<http://geneontology.org/>). We used either panther generic mapping package (<http://pantherdb.org/>) or Kyoto Encyclopedia of Genes and Genomes (KEGG) database (<https://www.genome.jp/kegg/pathway.html>) to examine the most enriched pathways. To compare the expression of the fibrogenic and angiogenic markers during human tendinopathy, data were downloaded from gene expression omnibus database (GEO dataset referenced: GSM26051).

Chipseq de novo motif analysis was performed using Homer (Hypergeometric Optimization of Motif EnRichment) software (homer.ucsd.edu/homer/).

# 9630015K15Rik coding potential was assessed by CPAT (Coding-Potential Assessment Tool) software (Wang et al., 2013) (http://lilab.research.bcm.edu/cpat/index.php).

Supplemental figures


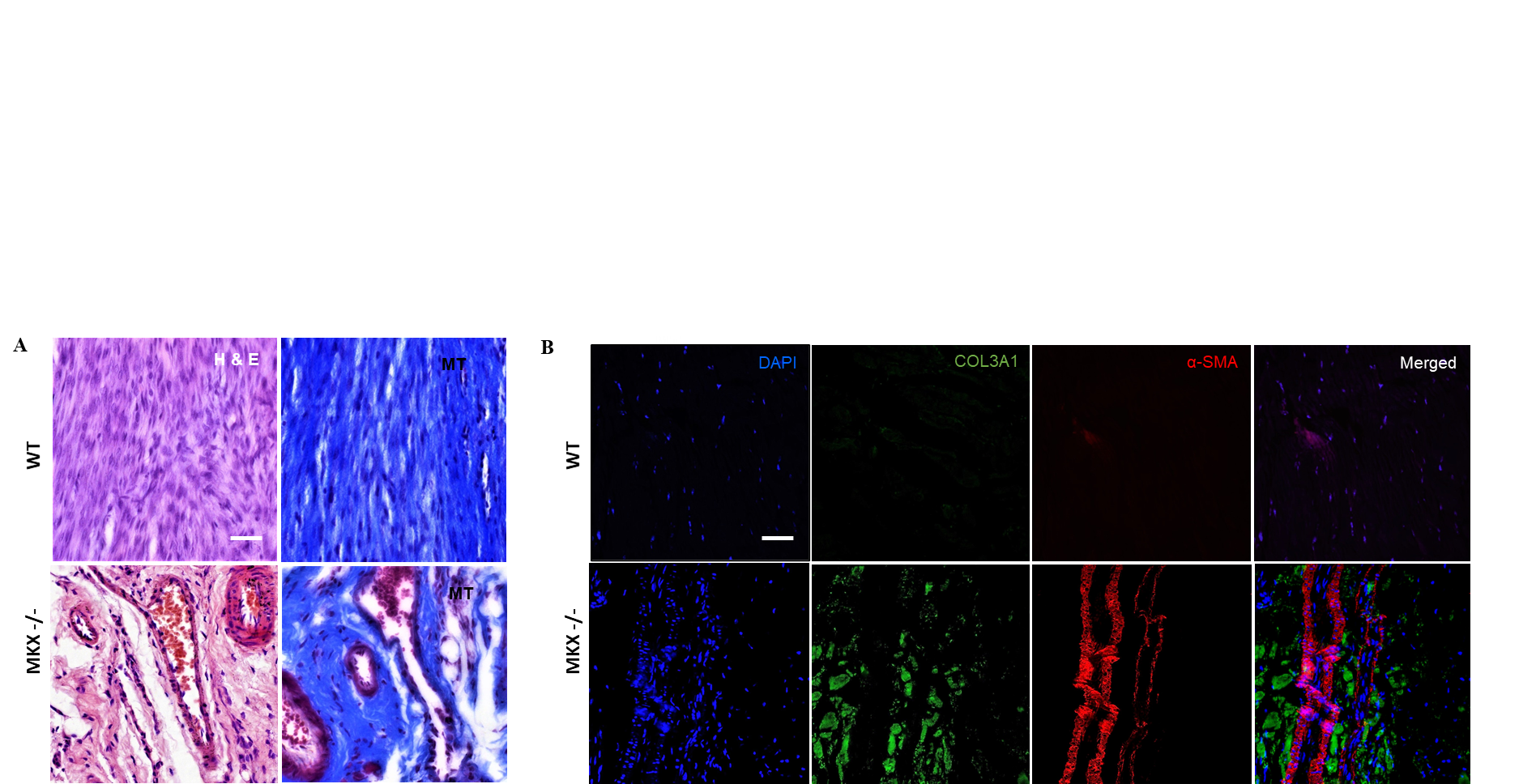


**Supplementary Figure 1:** **Fibrovascular scar formation study during neonatal regeneration. A.** Representative images of H&E and Masson Trichrome staining of Achilles tendon sections isolated from WTand *Mkx* Ko mice 28 days after injury (*n* = 3 per group). Scale bars, 40 μm. **B.** COL3A1 and α-SMA immunofluorescence staining of transectioned *Mkx* KO neonatal Achilles tendons vs. WT (representative images, N=3 per group). Scale bar, 40 μm.

**
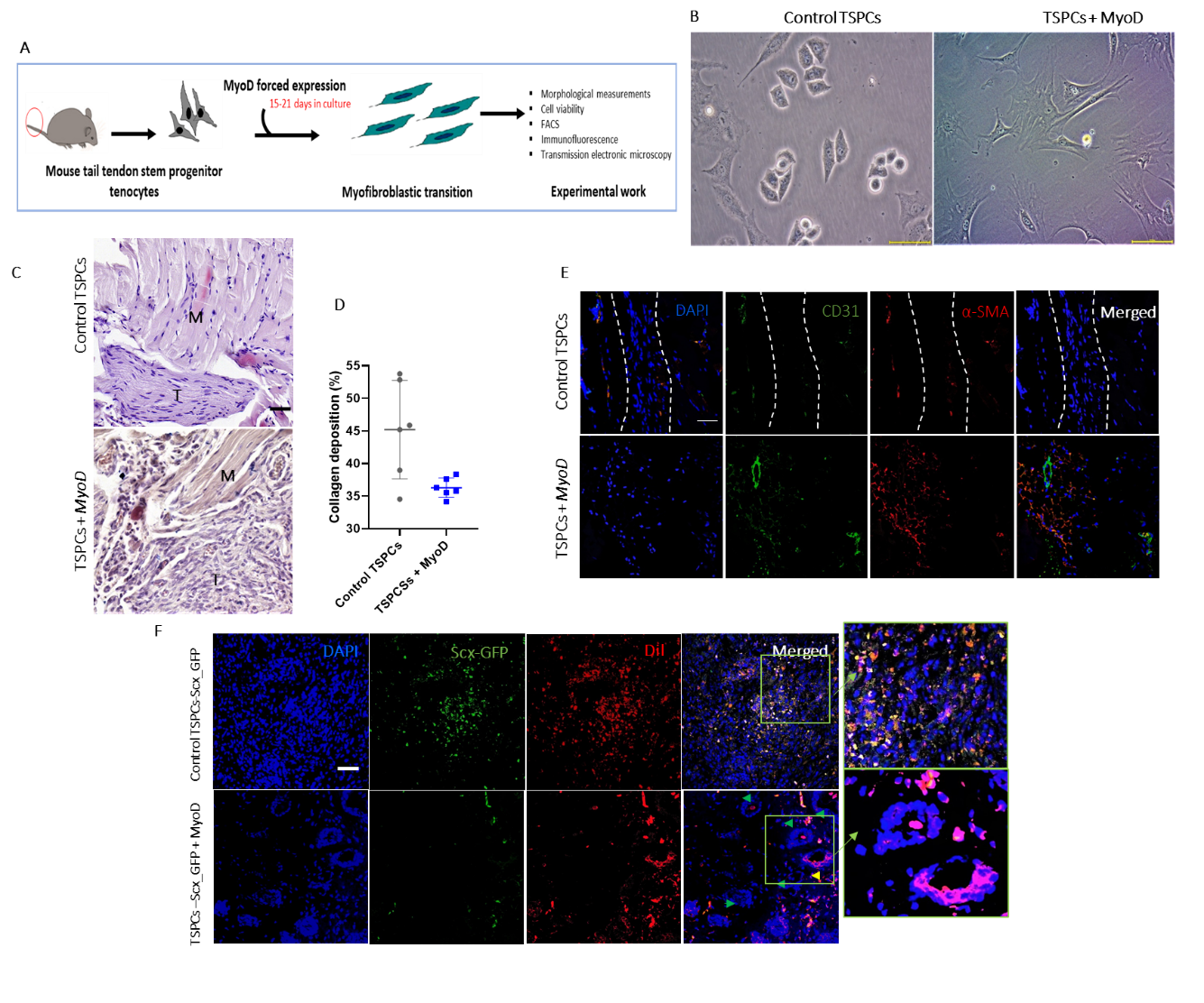
**

**Supplementary Figure 2.** **Myofibroblasts promotion of fibrovascular scarring *in vivo*.**

**A**. Schematic representation of the experimental design. **B.** Filopodia formation 7 days after MyoD transfection. Scale bar, 50 μm. Representative images of H&E and Masson’s trichrome (**C)** and Collagen deposition quantification **(D)** of stained sections of control TSPCs transplant (showing tendon-like tissue) or myofibroblasts transplant (fibrovascular scarring) 28 days after surgery (n = 5 per group, M: muscle; T: tendon). Scale bar, 50 μm. **E.** Immunoflorescence staining of CD31 and α-SMA in control or myofibroblast transplants (d28 after surgery, n=5), Scale bar, 40 μm. Dashed lines indicate tendon-like structures. **F.** Confocal microscopy images of Dil-stained MyoD-expressing TSPCs for 14 days or control TSPCs (d28 days after transplantation, n=6). Scale bar, 50 μm.

**Supplemental table**

| Skeletal muscle  markers |  | m-MyHC-F | GCATCGAGTGGACCTTCATTGAC |
| --- | --- | --- | --- |
|  |  | m-MyHC-R | ACAGTCTCATTGAGAGGGTCCTTG |
|  |  | m-Myog-F | ACGAGCGGACTGAGCTCAGCC |
|  |  | m-Myog-R | GGTAGCGGAGGTCCCGCTCCTCCTGGTTGAG |
|  |  | m-Dmd-F | GATTCTCCTGAGCTGGGTCCGAC |
|  |  | m-Dmd-R | GCCTTGGCAACATTTCCACTTCCTG |
| Fibrotic markers |  | m-Acta2-F | GTCCCAGACATCAGGGAGTAA |
|  |  | m-Acta2-R | TCGGATACTTCAGCGTCAGGA |
|  |  | m-Col3a1-F | AGGTGGACCAGGCAATGATGGAAA |
|  |  | m-Col3a1-R | TTCCTTTAGGACCAGGGAAACCCA |

**Table S1.** Primers used for qRT-PCR analysis

**Supplementary references**

Livak, K.J., and Schmittgen, T.D. (2001). Analysis of relative gene expression data using real-time quantitative PCR and the 2(-Delta Delta C(T)) Method. Methods San Diego Calif *25*, 402–408.

Wang, L., Park, H.J., Dasari, S., Wang, S., Kocher, J.-P., and Li, W. (2013). CPAT: Coding-Potential Assessment Tool using an alignment-free logistic regression model. Nucleic Acids Res. *41*, e74–e74.
